# Hand size estimates of fibromyalgia patients are associated with clinical and experimental pain

**DOI:** 10.1371/journal.pone.0270701

**Published:** 2022-07-25

**Authors:** Roland Staud, Rachel Carpenter, Melyssa Godfrey, Michael E. Robinson

**Affiliations:** 1 Department of Medicine, University of Florida, Gainesville, FL, United States of America; 2 Department of Clinical and Health Psychology, University of Florida, Gainesville, FL, United States of America; Anglia Ruskin University, UNITED KINGDOM

## Abstract

**Introduction:**

Simply inspecting one’s own body can reduce clinical pain and magnification of body parts can increase analgesia. Thus, body perceptions seem to play an important role for analgesia. Conversely, pain may also affect bodily perceptions. Therefore, we evaluated the effects of clinical and/or experimental pain on perceived hand size in fibromyalgia patients (FM) and healthy controls (HC).

**Methods:**

To investigate the effects of chronic and/or acute pain on size perception we compared hand size estimates of 35 HC and 32 FM patients at baseline and during tonic mechanical pain stimuli applied to one ear lobe. Mechanical stimuli were adjusted for each individual pain sensitivity to achieve a rating of 4 ± 1 VAS (0–10) units. Photographs of each subject’s hands were digitally manipulated to produce a monotonic series of 5 images larger and 6 smaller than actual size which were then presented to the participants in ascending and descending order (total number of images: 12).

**Results:**

FM and HC participants’ clinical pain ratings at baseline were 3.3 (3.1) and .3 (.8) VAS units, respectively. At baseline, FM participants selected significantly smaller hand images than HC as representative of their actual size (p < .02). During application of tonic experimental pain, the image size chosen to represent their actual hand size decreased significantly in FM participants and HC (p < .001) but this decrease was not different between groups (p > .05). Hand size estimates of FM participants correlated negatively with their clinical pain ratings (p < .04).

**Conclusion:**

The decreased hand size perception of FM patients and HC was associated with their clinical and/or experimental pain, supporting the hypothesis that pain can result in visual body distortions.

## Introduction

In the past, the body size-perception in healthy individuals was thought to be highly accurate, and as such it was used as a standard in early studies to interpret body size misperceptions in ill persons [1]. Subsequent studies, however, called this assumption into question, as evidence in healthy individuals showed systematic distortions of body size estimations. When in previous studies healthy participants were asked to judge the length of different body segments, a pattern of length mis-estimation was identified, suggesting that some body parts including the arms are misperceived as longer compared to others parts like the head and leg. Importantly, a pattern of misperception was found related to a body part’s representation in the primary somatosensory cortex (SI). For example, body parts like the hand, with their large cortical presence in SI, were estimated to be shorter, possibly compensating for their large somatosensory representation [2, 3]. Furthermore, body size estimates seem to be affected by additional factor, including pain [4–9].

Body distortions seem to be particularly relevant for post-amputation pain. After limb amputations approximately 80% of patients experience a body distortion known as phantom limb [10, 11]. The missing limb is often perceived as telescoped and shrunk with the proximal portion of the limb shortened and the more distal portion located near the stump [12]. Such body distortions are often associated with pain and structural remodeling of the central nervous system (CNS) [13], specifically with the reorganization of the primary somatosensory cortex (S1), which seems to correlate with the phantom limb experience. It appears that the perceived size of the phantom limb depends on its topical representation in S1. Such CNS reorganization seems to occur quite rapidly, as deafferentation of a body part appears to promptly reduce the activity of somatotopic region of S1 responding to the body part [14]. Regarding body sized distortions in patients with chronic musculoskeletal pain, only limited information is available. Most of this information comes from studies of patients with complex regional pain syndrome (CRPS) whose clinical presentation is often associated with tissue alterations of the affected painful extremity [15, 16]. Complex regional pain syndrome (CRPS) also involves changes of S1 representation of the affected limb [17] and is often associated with the perception of marked swelling of the extremity. Although the distortion of body images may be an important part of the presentation of CRPS, the mechanisms involved are not clear.

There is only limited data concerning cortical reorganization in patients with back pain. In some reports patients with back pain report their back as swollen when it is not or report difficulty in feeling their back [18, 19]. Overall, abnormal ongoing input from the periphery seems to be involved in the increased S1 representation of affected body areas [20]. Regardless of contributing factors, a change in intracortical inhibition is probably the common mechanism. This may involve unmasking of latent excitatory synapses, increased density of postsynaptic receptors, changes in conductance of the neuronal membrane, decreased inhibitory inputs, or the removal of inhibition from excitatory inputs [21]. Overall, the associations of acute and chronic pain with perceived body size estimates are only partially understood.

Different from most other chronic pain disorders, fibromyalgia syndrome (FM) is characterized by widespread pain, fatigue, insomnia, and polysymptomatic distress [22]. Most FM patients report painful body areas in the neck, shoulders, and low back and the number of their pain areas correlates with clinical pain [23, 24]. Because no specific peripheral tissue abnormalities have been detected in FM that could explain patients’ symptoms, CNS abnormalities, including dysfunctional pain modulation, have been implicated as FM mechanisms [25, 26]. However, additional factors that contribute to FM pathogenesis, need to be considered. Up to 70% of FM patients complain of unspecific distortions of their extremities, mostly their hands [27, 28]. Despite extensive laboratory testing and x-ray examinations, self-reported hand distortions of FM patients most often remain unexplained [29, 30]. In addition, FM patients report many symptoms, including poor balance and frequent falls which may indicate disturbed sensorimotor function and altered body perception [29, 30]. In addition, FM patients seem to perceive their total body size as altered during exacerbations of pain [31], suggesting a relationship between distortions of their body and pain. Using the Body Image Scale [32], a possible relationship between poor body image and severity of clinical FM pain has been previously reported [33].

Research on perceptual distortions is important, given that one may speculate that body pain might contribute to the initiation and maintenance of perceptual alterations and ultimately such knowledge may create better understanding of chronic pain states like FM [34]. Because FM patients frequently report many body parts as distorted together with widespread musculoskeletal pains and soreness [35] that include the extremities, we hypothesized that a) hand size estimates of FM patients would be abnormal at rest compared to normal pain free controls (HC), and that b) the variability of perceived hand size would be related to FM patients’ clinical pain.

## Methods

### Participants

Participants were recruited from the local community and FM support groups. All participants provided written informed consent to participate in this study. The University of Florida Institutional Review Board approved the procedures, including written informed consent procedures, and protocol for this study and all procedures of the study protocol conformed to the ethical guidelines of the 2013 Declaration of Helsinki. Prior to testing, all participants underwent a clinical examination by the physician investigator (RS) and were excluded from the study if they had significant abnormal physical findings unrelated to FM, specifically neurological abnormalities like peripheral neuropathy. Chronic conditions like hypertension and thyroid disease were permitted if they were well controlled by medical therapy. Patients could have conditions co-morbid with FM, like irritable bowel syndrome, chronic headaches, or temporo-mandibular disorder. All participants were asked prior to the study procedures whether they perceived their hand size as abnormal.

### Inclusion and exclusion criteria

Inclusion criteria for participants were 1) adults over the age of 18; 2) the ability to give informed consent; and 3) HC participants had to be healthy; FM patients had to fulfill the 1990 American College of Rheumatology Criteria for FM including wide-spread pain [36]. 4) Normal neurological examination, 5) normal or corrected-to-normal eyesight.

Exclusion criteria were 1) a relevant medical condition besides FM; 2) current participation in another research protocol that could interfere or influence the outcome measures of the present study; 3) the inability to give informed consent; 4) a recent non-healed hand injury; 5) chronic pain in hands; 6) current use of analgesic drugs, anxiolytic drugs, anti-depressants, or cough suppressants. All participants taking analgesic drugs or antidepressants before enrollment were asked to complete a wash-out phase (5 half-lives) prior to study entry.

### Ratings of experimental and clinical pain

A 15 cm mechanical visual analogue scale (VAS; 0–10) was used for ratings of experimental and clinical pain [37]. This scale is anchored on the left with “no pain at all” and on the right with “the most intense pain imaginable”.

Before experimental pain induction, participants were instructed to rate the experimental pain intensity at the start and end of mechanical stimulation using the VAS.

### Digital hand size manipulations

**Fig 1:**
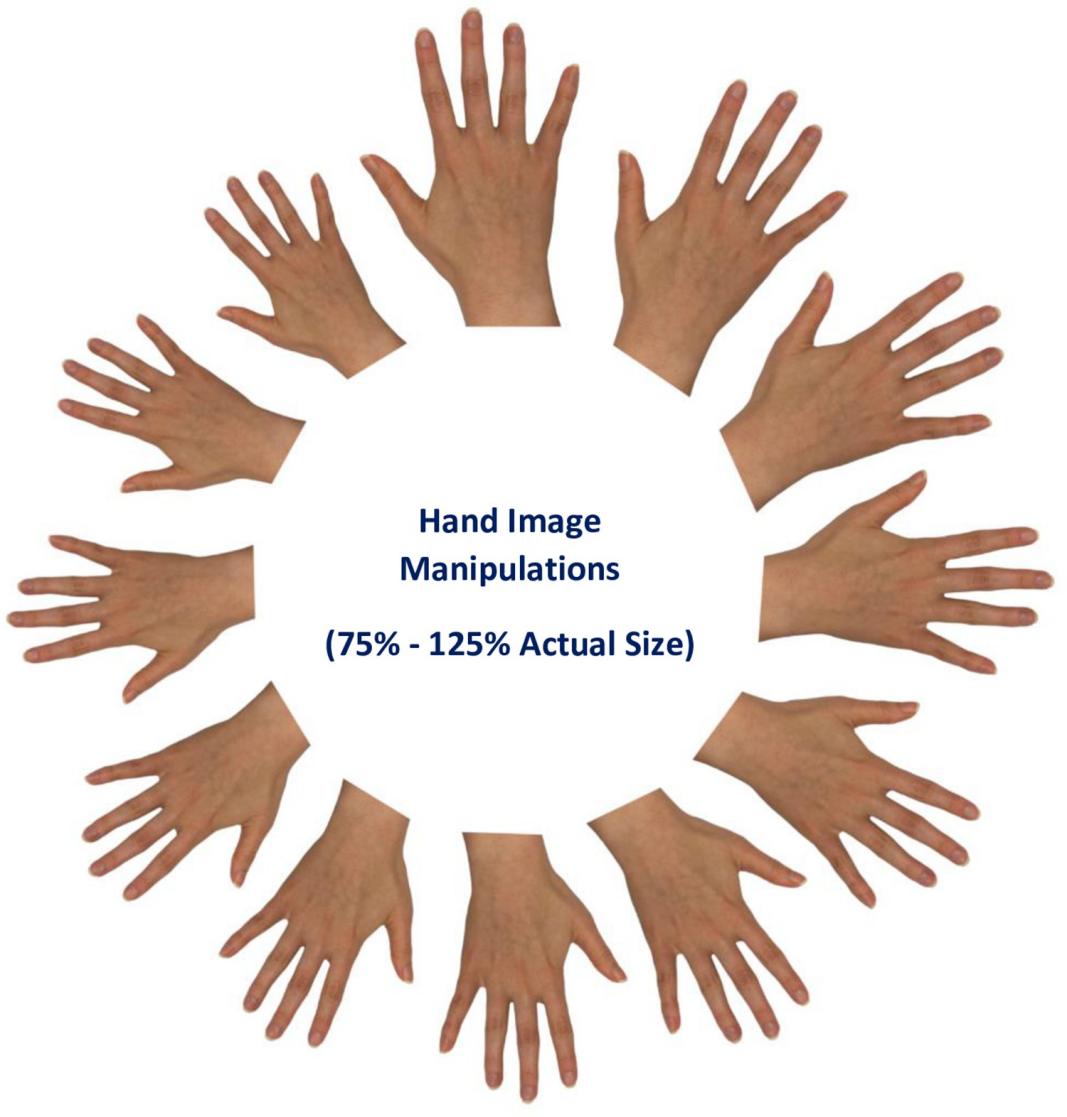
Example of image manipulations of study participants’ non-dominant hand. A high-resolution photograph of each participant’s hand was computer manipulated to provide images between 75% and 125% of actual size. Twelve hand images were presented to the participants in ascending and descending order on a computer screen at a pre-specified distance while their own hands remained hidden. The participants were asked to identify the image that was identical in size to their own hand.

Participants placed their non-dominant hand palms down on a flat surface and digital images of the dorsal aspects of the hand were taken using a digital camera (Powershot A1, Canon.com) mounted on a tripod. All jewelry was removed from the hands before 2 flash photographs of the non-dominant hand were taken resting palm down on a white background. The camera was positioned 19 centimeters above the non-dominant hand. Based on image quality the best image was selected for size manipulations and used in subsequent slide presentations. First, the image was edited using GIMP2.10.4 (Gimp.org) to provide a uniform, white background without any shadows. Second, the images were scaled in 4.3% steps from 74% to 122% of actual hand size (Fig 1). The actual size hand image (100%) was displayed on the monitor and by placing their hand over it each subject verified their hand image as a true match.

Using these images, a computer screen presentation was created that contained 4 sets of 12 images. The images were presented in ascending and descending order and the order of presentation of all images was counterbalanced for each individual and the same number of smaller or larger images was displayed. Each image was presented for 3 s. During the presentation all participants were asked to verbally identify the image that most closely matched the size of their own hand.

### Experimental design

#### Hand image presentation

*A) Hand size estimates at baseline*. Prior to the presentation of various size hand images, the participants rated their current clinical pain intensity using the VAS. The resolution of the computer screen was set to 1280 by 1024 pixels with 32-bit color quality on a 20-inch computer monitor positioned 23 inches from the seated subject’s forehead with the participant’s hands out of sight under the desk. All participants were told that they would see images of their own hand including images smaller and larger than actual size, but also actual size hand images. Furthermore, these images would be presented in ascending and descending order in 4 sets of 12 and a lab assistant would verbally assign each image an ascending or descending number between 1–12. After each set, each participant was then asked to identify one of the 12 images that most perfectly corresponded to the actual size of their own hand, i.e. if they put their hand on the computer screen, it would be an exact match with the screen image. The participants were also told to treat each set of 12 images like it was a completely different set and that they would be notified whenever a new set was beginning. Four image sets were shown and one image each was selected by every participant. A mean hand size estimate of each subject was obtained from the selected images.

*B) Hand size estimates during tonic pain*. To find out whether the variability of hand size distortions is associated with afferent input from areas other than the hand, we applied tonic pressure to the earlobe to induce tonic pain. For this purpose, a plastic spring-loaded pressure device with a surface area of 1x.5 cm was placed on the ear lobe corresponding to the non-dominant hand. This area has been found suitable for the induction of pressure pain in human participants [38]. The pressure delivered by the device was adjusted to achieve a target pain rating of 4 ± 1 VAS units. If the initial pain ratings were too high or too low, device pressure was adjusted to achieve the desired pain level of 4 ± 1 VAS units. Subsequently, the participants were asked to watch 4 sets of randomized hand image presentations using the same instructions as before. Hand size estimates after each of 4 sets were recorded. Before the ear device was removed, pain ratings were obtained to confirm that pain of 4 ± 1 VAS units had been maintained throughout tonic pain experiment. After the pressure device was removed overall pain intensity ratings were again obtained using the VAS. The participants were asked to keep their hands hidden throughout all slide presentations and to maintain the appropriate distance from the computer screen.

#### Tender point testing

As part of the FM classification, nine paired tender points (TP) as defined by the American College of Rheumatology 1990 FM Criteria [36] were assessed in all study participants by a trained investigator using the Wagner dolorimeter. TP are used to assess pressure pain sensitivity on muscles and tendon insertions at the trunk and extremities. The dolorimeter was placed on the examination site and the tissue pressure was gradually increased by 1kg/s until the participants reported it as painful. The participants were instructed to immediately report when the sensation at the examination site changed from pressure to pain by saying “now”. Pressure testing was stopped at that moment and the result were recorded as positive (1) if maximal pressure was ≤ 4 kg. If no pain was elicited at ≥ 4 kg the test results were recorded as negative (0) [36]. The presence of ≥ 11 positive TP (out of 18) is required for the 1990 classification of FM [36].

### Data analysis

Statistical analyses were calculated using SPSS 26.0 software (IBM, Inc.). Clinical pain, age, and TPs of participants were analyzed using independent t-tests. Histograms and the Kolmogorov-Smirnov test were utilized to confirm the normality of the data. A One-Sample t-test were used to test the difference of the hand-size estimates of the participants from the correct estimate. The effects of experimental pain on hand size estimates were tested with mixed ANOVA with group (FM, HC) and condition (baseline, tonic pain) as independent factors and hand-size estimates as dependent factor. For correlational analyses of clinical pain and hand-size estimates, Pearson’s product-moment was used. A threshold of < .05 was considered significant.

## Results

We enrolled 35 HC and 32 FM participants into the trial. 1 HC and 2 FM participants were male. The mean (SD) age of HC and FM participants was 47.2 (11.6) (range 20.6–60.2) and 52.8 (9.3) (range 24.1–64.3) years, respectively (p > .05) (Table 1). The mean (SD) number of tender points was significantly different between FM participants [17.1 (1.5)] and HC [3.4 (4.2)] (t = p < .03). All participants reported the size of their non-dominant hand as “normal” (not distorted and not swollen) and without pain at the beginning and end of the study.

**Table 1:** Characteristics of study participants.

	HC	FM	p-Value
**Age (years)**	47.2 (11.6)	52.8 (9.3)	> .05
**Clinical Pain (0–10)**	.3 (.8)	3.3 (2.1)	< .001
**Tonic Pain Start (0–10)**	3.4 (.7)	3.6 (1.2)	> .05
**Tonic Pain End (0–10)**	3.5 (1.5)	3.7 (1.9)	> .05
**Tender Points (0–18)**	3.4 (4.2)	17.1 (1.5)	< .03

### Clinical and experimental pain ratings of participants

The mean (SD) clinical pain ratings of HC and FM participants at baseline was .3 (.8) and 3.3 (2.1) VAS units, respectively (t = – 8.1; p < .001). The mean (SD) experimental pain ratings of HC and FM patients at the ear at the start of the experiments was 3.4 (.7) and 3.6 (1.2), respectively (t = .2; p > .05). These pain ratings did not significantly differ from those obtained at the end of the experiment in HC [3.5 (1.5)] and FM participants [3.7 (1.9)] (t = 2.0; p > .05).

### Matching hand images at baseline and during experimental pain

**Fig 2:**
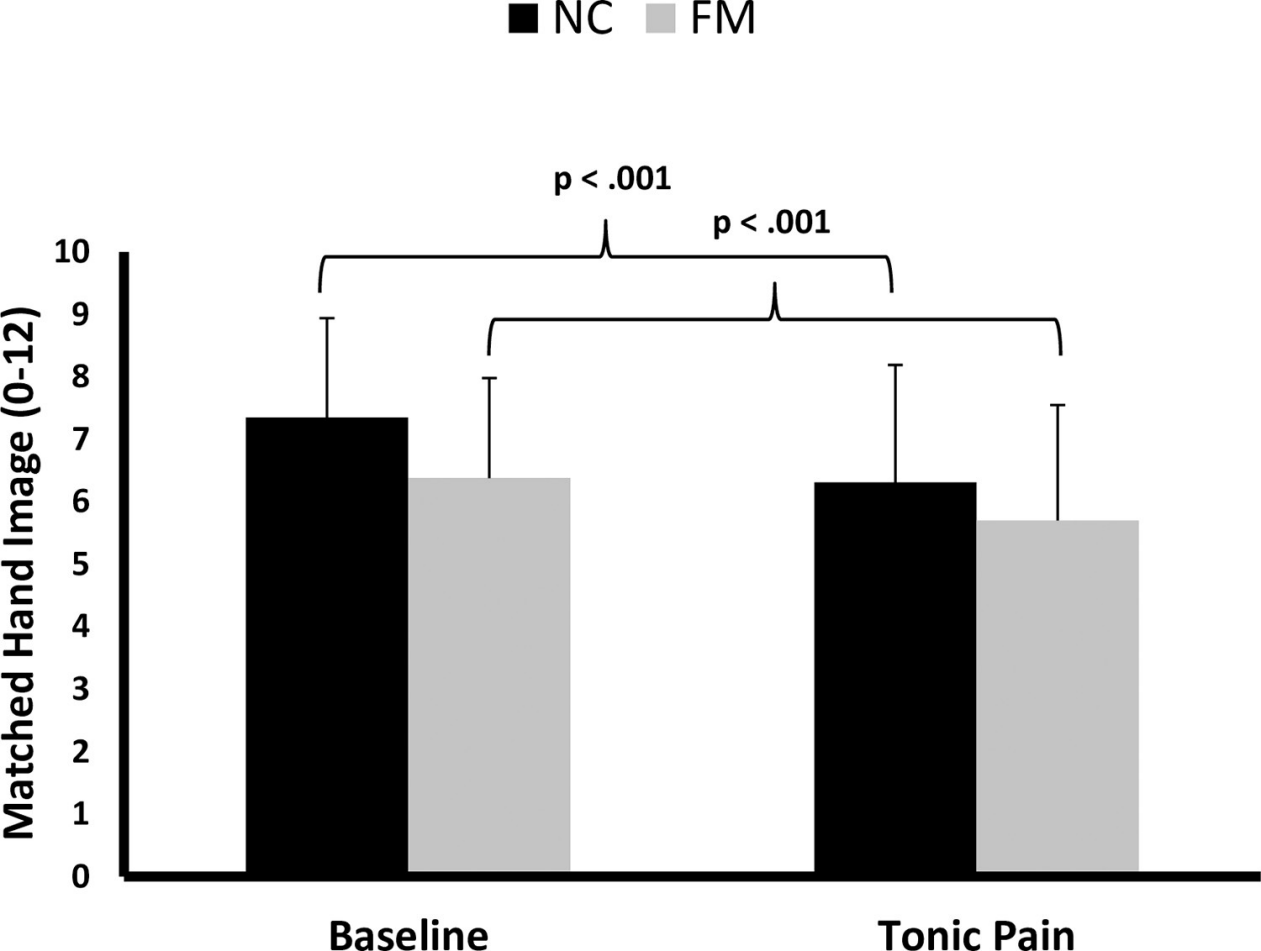
Average (SD) matched hand images by FM participants and HC at baseline and during tonic mechanical pain. While matched hand images of FM participants were significantly smaller than HC at baseline, both groups’ image sizes decreased during application of tonic mechanical pain (p < .001). This change in matched hand images was not significantly different between groups (p > .05).

Prior to the experiments none of the participants reported abnormal perception of their hand size. Of the 12 digitized hand images of different sizes shown to the participants, image 7 was the one displaying the accurate hand size. Thus, image numbers < 7 represented smaller than actual hand images, whereas image numbers >7 corresponded to larger than actual hand images. After reviewing 4 sets of images while seated in a chair, the mean matched image (SD) selected by HC and FM participants as representative of their actual hand size was 7.4 (1.6) and 6.3 (1.6), respectively (Fig 2). This difference from their true size was statistically significant for FM subject (t = -2.3; p = .03) but not for HC (t = 2.0; p > .05), indicating that FM subject significantly underestimated their hands size by 8.9% whereas HC did not. During tonic pressure pain at the ear lobes the image number selected by HC and FM participants as representing their actual hand size, decreased to 6.3 (1.9) and 5.7 (1.9), respectively. A mixed ANOVA with hand image as the dependent variable and diagnostic group and condition as independent variables showed significant main effects for condition (F(1,65) = 27.6; p < .001; part eta2 = .30) and diagnostic group (F(1,65) = 1,079.0; p < .05; part eta2 = .06). The interaction effect of condition x diagnostic group, however, was not statistically significant (F(1,65) = 1.2; p > .05; part eta2 = .02). This analysis demonstrated that hand images chosen by FM participants at baseline were significantly smaller compared to HC. Image sizes selected during experimental pain induction by both FM and HC participants were significantly smaller compared to baseline, however, this change did not differ statistically between groups.

### Correlation of clinical pain with matched hand images of FM participants

**Fig 3:**
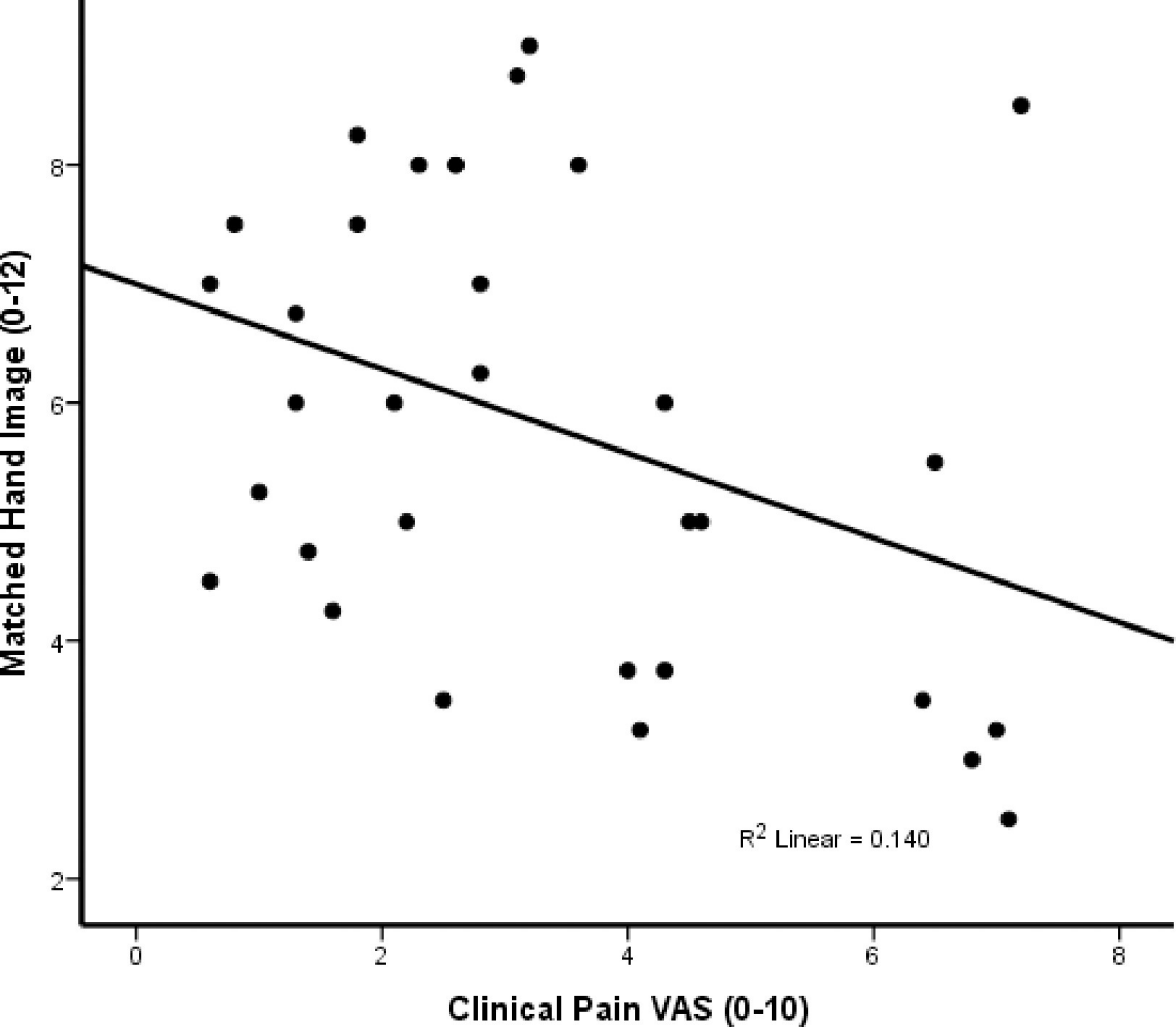
Pearson product moment correlations demonstrated a significant negative association between clinical pain of FM patients and their matched hand image size at baseline (p = .04). The perception of decreased hand size was associated with increased clinical FM pain.

Average (SD) matched hand image and clinical pain ratings of FM participants at baseline were 6.4 (1.6) and 3.3 (2.0) VAS, respectively (Fig 3). A Pearson Product moment correlation demonstrated a negative association between matched hand image size and clinical FM pain [r = -.378 (p = .04)], indicating that the perception of decreased hand size is associated with increased overall FM pain.

## Discussion

Our study showed that FM patients significantly underestimated their own hand size at baseline and this effect was correlated with their clinical pain (R^2^ = .14). In contrast, the hand estimates of HC nearly accurately matched their true hand size at baseline. Application of sensitivity-adjusted tonic mechanical pain stimuli decreased hand size estimates of HC and worsened those of FM patients, suggesting that clinical and experimental pain decrease visual hand size estimates in healthy individuals and FM patients. Importantly, the magnitude of pain related distortions of hand size estimates was similar in HC and FM participants, suggesting that this mechanism is not abnormal in FM patients.

Similar visual distortions of body parts have also been observed in other chronic pain conditions than FM such as low back pain [5], CRPS1 [15], and osteoarthritis [39] and cortical reorganization has been suggested as the basis for such distortions [40]. Such cortical changes seem to involve somatotopic changes of S1 [41] which also seem to occur with regional anesthesia. Previous studies have reported that regional anesthesia is associated with the experience of subjective swelling [42] and results in shrinkage of sensory cortex 1 (S1) representation of the affected area [43]. For example, complete anesthesia of the thumb by digital nerve block significantly increased its perceived size (by approximately 70%) in healthy individuals [43]. Similar effects were noted after application of topical anesthetics to the lips [43], indicating that the perceived size of body parts can change rapidly when afferent input to the central nervous system is altered. Conversely, S1 representation of body areas may increase during clinical and experimental pain and result in decreased size estimates as seen in our study. Furthermore, such cortical reorganization appears to be highly efficient, as the percept of FM and HC participants rapidly changed during application of experimental pain stimuli. Although it appears that body size-perception can change quickly after deafferentation, they do not become erased, even after sensory input to the cerebral cortex is interrupted following amputation or spinal cord transection [44]. Whereas previous research suggests that the experience of chronic pain in a specific body areas is associated with perceptual and sensorimotor disturbances of the affected body part [45, 46], our study of HC and FM patients showed that painful stimulations in one location can result in distortions of the percept of other body areas. Whether this distortion is associated with similar cortical changes than seen in other chronic pain syndromes, is unknown at this time. However, several MRI studies of FM patients demonstrated decreased gray matter of the somatosensory cortices which could be associated with altered body size perception [47, 48]. Overall, pain and anesthesia seem to have opposite effects on body size estimates of human participants and the central nervous system mechanisms underlying these effects seem to involve neural plasticity of cortical areas related to body schemas. Although we did not specifically investigate the neural changes associated with experimental pain, they do appear to be similar in healthy individuals and FM patients. At this time, it is unknown if such cortical changes are similar across most musculoskeletal pain conditions or if they depend on duration and type of each specific pain disorder. Whether the perceived size reduction of FM patients’ hands associated with clinical pain is due to plastic changes of S1 or other brain areas needs to be addressed in a future investigation.

Not all studies, however, have reported the same association between pain and decreased body size estimates in human participants. For example, a study of patients with complex regional pain syndrome 1 (CRPS1) reported that estimates of patients’ affected limb size were not smaller but slightly enlarged (107% of actual size) [15]. Because CRPS1 has been associated with shrinkage of S1 representation of the affected body part [17], it has been speculated that this change increases the visual percept of the affected body area similar to that seen with regional anesthesia [43]. However, the perceived size increase of body areas in CRPS1 patients did not correlate with their clinical pain [15]. It is possible that shrinkage of the S1 representation of the affected body area involves alteration of visual magnitude perception. Interestingly, the perceived size of the affected limb was related to the duration of CRPS1 [15]. Several other studies seemed to indicate that perceived body size may depend not only on pain or anesthesia. For example, one study of HC reported a small but significantly enlarged perception of thumb size during cold stimuli [43] while another study demonstrated that anesthesia of the whole arm created the percept, that it was shortened and closer to the body [44]. A possible explanation for these findings is that the increase in perceived size with anesthesia may be related to rapidly enlarging receptive fields of cortical neurons [49, 50] and driven by increased basal activity of central neurons which normally process sensory input from the anesthetized area [51]. The results of such central changes may be the perceptual increase of the size of a body part. On one hand, it appears that pain related perceived increase in body areas depend on the size of the deafferented body part [52, 53]. On the other hand, when large limb areas become deafferented after amputation, the extremity is frequently perceived as shorter and painful (phantom limb) [44]. This process is gradual and occurs over time and it is possible that the frequently reported chronic pain after amputations is related to the perceived shrinkage of phantom limbs.

It is possible that not only pain but also nociceptive sensitivity may be enhanced by changes in the body-image. A number of investigators have shown that sensitivity to experimental pain is increased when perception of the body part is distorted by visual manipulation [54–56], and clinical disruption of perceptual awareness might have a similar outcome in patients with phantom limbs.

Whether biased visual attention to painful body areas plays an important role for body size distortions in patients with chronic musculoskeletal pain is not well understood. However, there is at least some evidence against this assumption from a study of patients with CRPS [57]. In this study the effect of experimental and chronic pain on visual attention of CRPS patients was evaluated. The results showed that unilateral, continuously applied pain stimuli or chronic pain had no or only very limited influence on visual attention.

### Limitations

Our study demonstrated robust effects of clinical and tonic experimental pain on perceived hands size but we used nociceptive manipulations only at the ear and not in other locations. Therefore, we cannot generalize our findings as effects of nociception, because distortions of body size-perception during the application of pain stimuli may differ depending on location. Although we tested only hand size estimates of study participants, given the widespread nature of FM pain, it appears possible that clinical and experimental pain impact estimates of other body areas as well. Future studies utilizing manipulations of nociception and analgesia will be necessary to explore the role of afferent input for size distortions of FM patients’ body areas. Furthermore, because we only investigated the effects of pain on body size perception, additional factors, including distractions need to be considered, as they may have influenced our results.

## Conclusions

Our study seems indicate that visual body size perception can be altered by clinical and experimental pain. FM patients underestimated the size of their hands at baseline and this effect was significantly correlated with their clinical pain. Application of experimental tonic pain stimuli further increased this effect. In contrast, HC’s estimates of their hands size were without significant distortions at baseline conditions, but during application of sensitivity adjusted pressure pain stimuli, their hand size estimates became significantly smaller. The magnitude of these pain related changes of size estimates was similar in FM and HC participants. Whether these pain related size distortions of HC and FM patients are limited to the hands or also involve other body areas needs to be addressed in future studies.
